# The effect of body size and composition on lumbar spine trabecular bone score in morphologically diverse subjects

**DOI:** 10.1371/journal.pone.0287330

**Published:** 2023-07-03

**Authors:** Jadwiga Malczewska-Lenczowska, Olga Surała, Dariusz Sitkowski, Beata Szczepańska, Maciej Zawadzki

**Affiliations:** 1 Department of Nutrition Physiology and Dietetics, Institute of Sport–National Research Institute, Warsaw, Poland; 2 Department of Physiology, Institute of Sport–National Research Institute, Warsaw, Poland; 3 High School of Rehabilitation, Warsaw, Poland; University of Life Sciences in Lublin, POLAND

## Abstract

**Aim:**

The trabecular bone score (TBS) is a tool for assessing bone quality and health. Current TBS algorithm corrects for body mass index (BMI), as a proxy of regional tissue thickness. However, this approach fails to consider BMI inaccuracies due to individual differences in body stature, composition and somatotype. This study investigated the relationship between TBS and body size and composition in subjects with a normal BMI, but with large morphological diversity in body fatness and height.

**Methods:**

Young male subjects (n = 97; age 17.2±1.0 years), including ski jumpers (n = 25), volleyball players (n = 48) and non-athletes (controls n = 39), were recruited. The TBS was determined from L1-L4 dual-energy X-ray absorptiometry (DXA) scans using TBSiNsight software.

**Results:**

TBS correlated negatively with height and tissue thickness in the L1-L4 area in ski jumpers (r = -0.516 and r = -0.529), volleyball players (r = -0.525 and r = -0.436), and the total group (r = -0.559 and r = -0.463), respectively. Multiple regression analyses revealed that height, L1-L4 soft tissue thickness, fat mass and muscle mass were significant determinants of TBS (R^2^ = 0.587, p<0.001). L1-L4 soft tissue thickness explained 27% and height 14% of the TBS variance.

**Conclusion:**

The negative association of TBS and both features suggests that a very low L1-L4 tissue thickness may lead to overestimation of the TBS, while tall stature may have the opposite effect. It seems that the utility of the TBS as a skeletal assessment tool in lean and/or tall young male subjects could be improved if tissues thickness in the lumbar spine area and stature instead of BMI were considered in the algorithm.

## Introduction

The Trabecular Bone Score (TBS) is a textural parameter which provides an indirect evaluation of trabecular microarchitecture in the lumbar spine (L1-L4) [1]. Existing data indicate that a low L1-L4 TBS is associated with both a history of fracture and occurrence of new fractures in the future [2]. Because TBS is independent from bone mineral density (BMD) in bone fragility evaluation [3], it offers a valid and complementary tool to improve bone health assessment [1, 2] and thus, has been included in the WHO Fracture Risk Assessment Tool (FRAX) [4].

TBS is derived from pixel grey-level variation in the lumbar spine (2D) DXA image. The texture of the DXA images depends not only on different X-ray absorption characteristics of regional bone, but also on body size and composition. The presence of soft tissues leads to X-ray attenuation, affecting the detection of grey-level variations, which may eventually alter the TBS estimate [5]. To overcome this error, the TBS algorithm considers body mass index (BMI) as a surrogate of regional soft (mainly fat) tissue thickness. Nevertheless, there is evidence that BMI may not be a sufficiently specific proxy and its clinical limitations might affect the TBS estimation [5–7]. For example, BMI does not distinguish between excess fat and muscle mass, nor does it provide any information on body size or fat distribution. Consequently, BMI might misclassify lean individuals with highly developed muscle as being overweight or obese, which often occurs in athletes.

Interestingly, there is data indicating that not only BMI and regional soft tissue characteristics, but also body stature [6, 7], might lead to biased estimates of the TBS. To date, studies investigating the possible effect of individual indicators of body stature and composition on the TBS are limited, ambiguous, and target only non-physically active adults [8, 9] and children aged 10–17 years [10]; hence, more research is needed to understand this relationship more completely, thereby informing algorithm development to overcome this bias.

The study aim was to investigate the relationship between TBS and body size and composition in male subjects, with particular emphasis on soft tissue in the L1-L4 area. To test the influence of body size and structure, as potential confounders in computing a TBS, we targeted lean adolescent subjects characterized with a normal BMI, but of highly diverse height and body composition. These conditions were met by representatives of two sports; volleyball (representing a very tall, slender body shape) and ski jumping (representing a lean body and average stature). We broadly hypothesized that body stature (height) and composition (soft tissues) would affect TBS estimation.

## Materials and methods

### Subjects

Ninety-seven young male subjects, aged 15–19 years, were recruited for this study. This cohort consisted of 25 ski jumpers (SJ) and 48 volleyball players (VP) who were members of national youth teams, and 39 non-athletes who formed a control group. The presented results are part of a more comprehensive project implemented in a group of physically active and untrained male youth. The exclusion criteria were: obvious signs of acute or chronic illness, which may affect bone health, a long period of immobilization or bone fractures in the previous 12 months, BMI >37 kg/m^2^ and <15 kg/m^2^ [11, 12]. The basic characteristics of studied groups are presented in Table 1. Biological age was estimated via bone age, based on activity of bone alkaline phosphatase (BALP).

**Table 1:** General characteristics of the study population (mean ± SD).

Variables	Ski jumping	Volleyball	Control	P value
Age (years)	17.0 ± 1.0	17.3 ± 0.8	17.3 ± 1.1	0.240
Body mass (kg)	58.3 ±7.2*	84.2 ± 9.2	77.3 ± 12.8	<0.001
Height (m)	1.74 ± 0.06	1.96 ± 0.07*	1.79 ± 0.08	<0.001
BMI (kg/m^2^)	19.9 ± 1.5*	21.9 ± 2.0	24.1 ± 3.6	<0.001
Mean vertebra area (cm^2^)	14.4 ± 1.5	18.2 ± 1.4*	15.0 ± 1.5	<0.001
Body type:				
• endomorphic	1.83 ± 0.48	2.42 ± 0.61	4.72 ± 2.42^	<0.001
• mesomorphic	3.46 ± 0.93	3.37 ± 1.09	4.55 ± 1.10*	<0.001
• ectomorphic	4.40 ± 1.12	4.27 ± 1.14	2.29 ± 1.63*	<0.001
BALP (μg/L)	65.1 ± 39.9	44.4 ± 18.9	45.1 ± 27.6	0.097
Training experience (years)	7.4 ± 2.4	6.5 ± 2.9	-	0.095

*–significantly different from the other groups

^–all groups differed from each other; BMI–body mass index, BALP–bone alkaline phosphatase

This study was carried out in accordance with the Declaration of Helsinki (2000) of the World Medical Association. The experimental protocol was approved by the Ethics Committee (KEBN-18-39-JM). Written informed consent was obtained from the subjects or their parents when the participant was under the age of consent.

### Procedures

#### DXA measurements

The DXA measurements were performed using the Lunar Prodigy DXA scanner (GE Healthcare Inc, Madison USA) and analysed using the GE Encore software (version 1.6). The DXA machine was calibrated at the start of each testing day using a quality assurance block and a spine phantom. The DXA scanner exhibited high reliability for the assessment of lumbar spine bone mineral content (BMC; CV = 0.6%), lumbar spine areal bone mineral density (aBMD; CV = 0.4%), and TBS (CV = 1.4%).

#### Lumbar spine BMD, BMC, and TBS measurements

In all subjects, aBMD (g/cm^2^) and BMC(g) were measured for the L1-L4 vertebrae. Age-matched Z-scores for the L1-L4 area were calculated with GE Encore software, using age-appropriate reference values. Since DXA-derived measurements of bone density provide details for area and not volume, L1-L4 bone mineral apparent density (BMAD) was calculated using a validated formula: BMAD = (L1 BMC + L2 BMC + L3 BMC + L4 BMC) / (L1V + L2V + L3V + L4V) [12]. The volume of each vertebra was calculated as the anterior-posterior bone area of the respective vertebra, raised to the power of 1.5.

Trabecular bone microarchitecture in the L1-L4 was assessed indirectly using TBS iNsight (Osteo) software (Version 3.0.2, Medimaps, Pessac, France). When the Z-score values for BMD in adjacent vertebrae differed by more than 1 SD, the outlier result was excluded, and the mean values of BMD, Z-score and TBS were calculated from 3 vertebrae [13]. In adults, values ≥1.350 are classified as normal, whereas values from 1.200 to 1.350 are recognized as partially degraded, and values <1.200 represent fully degraded bone microarchitecture [14].

#### Body composition

Body composition was measured in the morning (8.00–9.00 a.m.) using a bioelectrical impedance analysis (BIA) system (Tanita BC-420MA, Japan). Subjects were fasted, bladder voided, wearing only underwear, with all metal objects removed. The following body components were assessed: total fat mass (FM), fat-free mass (FFM), and muscle mass (MM), all expressed in kg and as a percentage. Data on L1-L4 tissue thickness (cm), as well as tissue fat percentage (%) in this region, were derived automatically from the DXA report.

#### Blood assays

Blood was obtained from an antecubital vein at 8.00–9.00 am, under fasting conditions, at least 38 h after the last exercise bout. Blood samples were collected into tubes containing coagulation accelerator and serum separator before centrifugation (10 min at 2000 × g). The serum aliquots were stored at -20°C for further tests of bone-specific alkaline phosphatase (BALP) activity. Sample testing was performed using a commercial enzyme immunoassay (IDS-iSYS CrossLaps Immunodiagnostic Systems Ltd, UK). The intra- and inter-assay CVs were 4.1% and 3.6%, respectively.

#### Anthropometric measurements

Body height (Seca 285, Germany), body mass and fat (Tanita BC-420MA, Japan), as well as waist circumference (cm) were assessed. For the latter, five skinfolds (triceps, subscapular, iliac crest, supraspinale, medial calf) were taken using a Harpenden skinfold caliper. All measurements were taken by the same researcher according to the ISAK [15]. Somatotype was determined using the Heath-Carter anthropometric method [16]. BMI was calculated as weight (kg) divided by height squared(m^2^), and waist-to-height ratio (WHtR) as waist circumference (cm) divided by height (cm).

#### Statistical analyses

Data distribution and homogeneity of variance were explored by the Shapiro-Wilk test and Levene’s test, respectively. Differences between groups were determined by a one-way analysis of variance with post-hoc comparisons using the Tukey test or Kruskal-Wallis test. Pearson correlations were used to explore associations between TBS and the different body size and composition variables.

To investigate which independent indices of body size and composition might predict the TBS, we performed a backwards stepwise multiple linear regression analysis on the pooled dataset (i.e., total group). To determine the relative importance of each predictor, the unique contribution of each variable was calculated using commonality analysis [17]. Alpha values of p<0.05 were considered statistically significant. Results are presented as means (±SD). Statistica 13.1 software (Dell Inc., Tulsa, OK, USA) was used for data analyses.

## Results

The studied groups did not differ in terms of chronological age, BALP activity or, in the case of athletes, training experience (Table 1). According to our assumptions, the groups differed significantly (p<0.001) in terms of height and fatness (endomorphic). Moreover despite similarities in the slenderness (ectomorphic) and muscularity (mesomorphic) of both athlete groups, they differed in terms of body mass and BMI, stature and mean area of vertebrae in the L1-L4 region (p<0.001).

### Bone tissue parameters

The TBS values were higher in ski jumpers (1.477±0.065; p<0.001) compared to both volleyball players (1.397±0.079; p<0.001) and controls (1.426±0.074; p<0.001). In contrast, volleyball players group had significantly higher (p<0.001) values of BMD and Z-score than the two other groups. However, after adjusting BMD for height (BMAD), the volleyball group differed significantly (p = 0.043) only from the control group (Table 2).

**Table 2:** Values of bone and anthropometric indices (mean ± SD).

Variables	Ski jumping	Volleyball	Control	P value
*LUMBAR SPINE L1-L4*				
BMC (g)	17.2 ± 3.0	25.5 ± 3.1*	17.7 ± 2.9	<0.001
BMD (g/cm^2^)	1.19 ± 0.15	1.40 ± 0.13*	1.17 ± 0.12	<0.001
BMAD (g/cm^3^)	0.36 ± 0.04	0.38 ± 0.04^&^	0.35 ± 0.03	0.043
Z-score	0.02 ± 1.0	1.45 ± 0.9*	-0.12 ± 0.85	<0.001
TBS	1.477 ± 0.065*	1.397 ± 0.079	1.426 ± 0.074	<0.001
*BODY COMPOSITION DXA*				
L1-L4 tissue thickness (cm)	17.3 ± 1.1*	20.2 ± 1.5	20.7 ± 1.1	<0.001
L1-L4 tissue % fat	5.0 ± 1.1	6.2 ± 1.7	16.9 ± 9.7^	<0.001
*BODY COMPOSITION BIA*				
FAT%	7.3 ± 2.4	10.8 ± 2.7	15.7 ± 5.9^	<0.001
Fat mass (kg)	4.4 ± 1.8*	9.2 ± 2.8	12.8 ± 6.4	<0.001
FFM (%)	92,7 ± 2.4	89.2 ± 2.7	84.3 ± 5.9^	<0.001
FFM (kg)	53.9 ± 5.8	75.1 ± 7.7	64.5 ± 7.3^	<0.001
MM (%)	88.0 ± 2.3	84.8 ± 2.6	80.1 ± 5.6^	<0.001
MM (kg)	51.2 ± 5.5	71.4 ± 7.4	61.3 ± 7.0^	<0.001

*–significantly different from the other groups

&–significantly different from Control group

^–all groups differ from each other; TBS–trabecular bone score; BMC–lumbar spine bone mass content; BMD–lumbar spine bone mass density; BMAD–lumbar spine bone mineral apparent density; FAT%–fat content; FFM- fat free mass; MM–muscle mass.

### Body size and composition indices

Tissue thickness in the L1-L4 area was significantly lower (p<0.001) in ski jumpers (17.3±1.07 cm) compared to the volleyball players (20.2±1.47 cm) and controls (20.7±1.07 cm) (Table 2). At the same time, significant between-group differences were found in the tissue fat content in this region (p<0.001) with tissue fat % being larger in controls (16.9±9.7%) than in volleyball group (6.2±1.7%) and SJ (5.0±1.0%). Relating to whole body composition, all groups differed (p<0.001) from each other in terms of fat mass percentage, as well as fat free mass and muscle mass expressed in kg and as a percentage. Body fat expressed in kg was significantly lower (p<0.001) in ski jumpers than volleyball players and controls.

### Correlations of TBS with age and body size and composition

**Fig 1:**
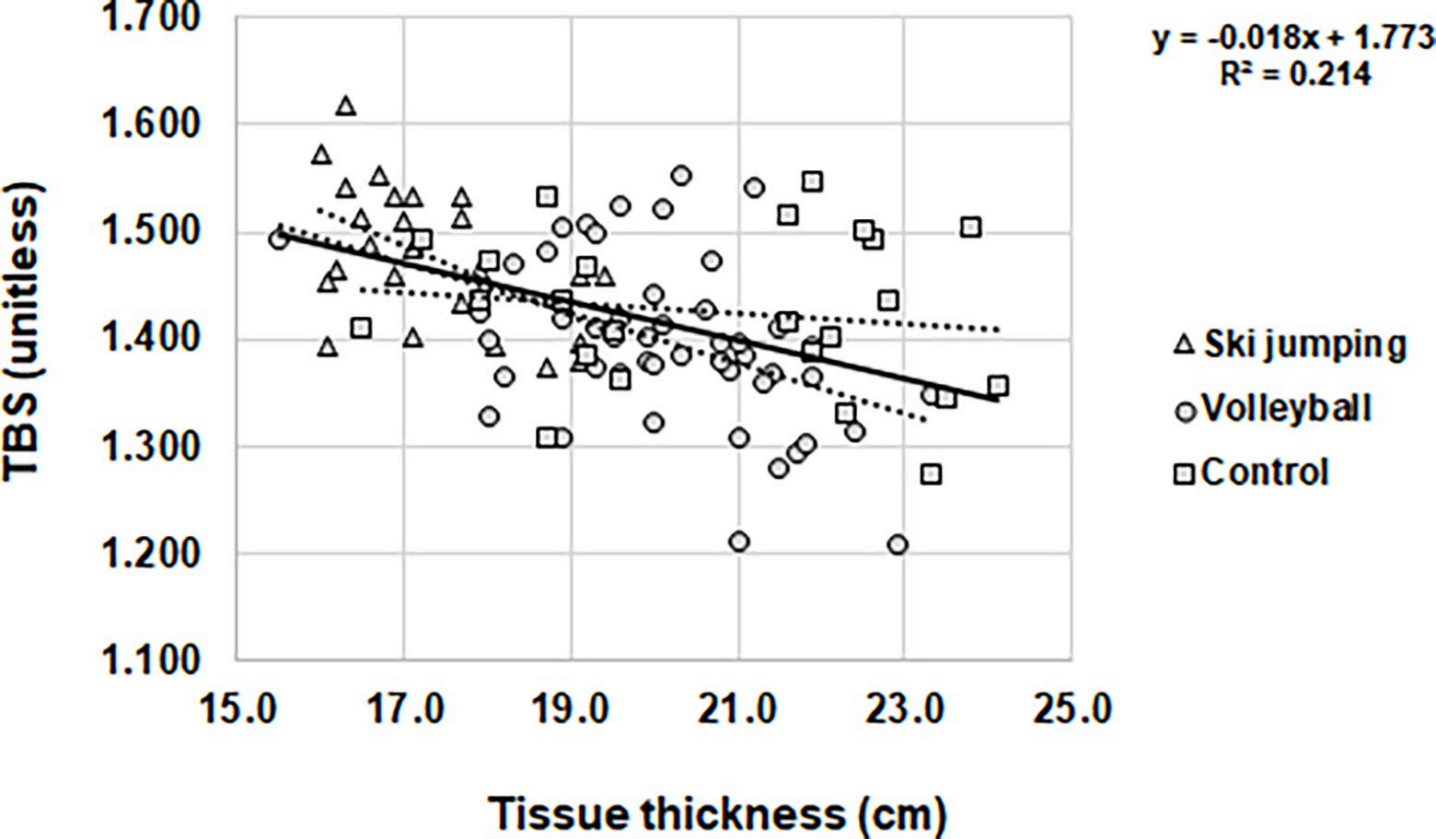
Relationship between trabecular bone score and L1-L4 soft tissue thickness in subgroups and in total group.

TBS correlated with height in both athletic groups (ski jumping r = -0.516; volleyball r = -0.525), and in the total group (r = -0.559) (Fig 1).

**Fig 2:**
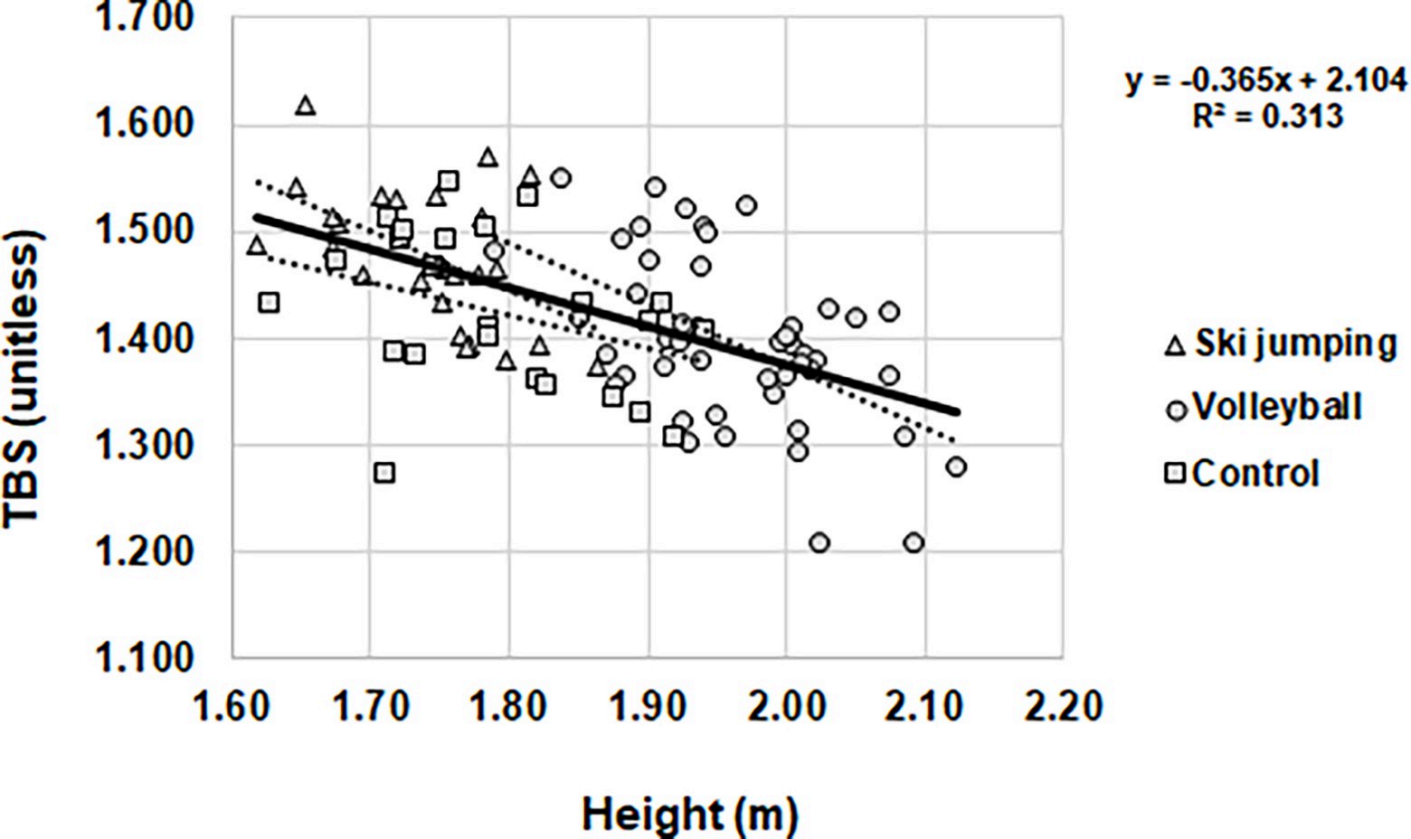
Relationship between trabecular bone score and height in subgroups and total groups.

Moreover, TBS correlated with mean vertebra area in all studied groups, both individually (ski jumpers r = -0.512; volleyball players r = -0.364 and controls r = -0.407) and in the total group (r = -0.522), as well as with L1-L4 regional soft tissue thickness in athletic males (ski jumpers r = -0.529; volleyball players r = -0.436) and the total group (r = -0.463) (Fig 2). Body mass correlated with TBS (r = -0.391), but only as a combined group.

Relationships with body composition also emerged (Table 3). TBS correlated significantly with fat free mass and muscle mass in volleyball group (r = -0.330 and r = -0.329, respectively) and the total group (r = -0.468 and r = -0.469, respectively). Conversely, the corresponding correlations were not significant (fat free mass = -0.380, p<0.061; muscle mass r = -0.386, p<0.057) among ski jumpers (Table 3).

**Table 3:** Pearson’s correlation coefficients between TBS, age and selected body size and body composition variables.

Group	Age (y)	Height (m)	Body mass (kg)	BMI (kg/m^2^)	WHtR		Total			DXA L1-L4 area		
						FAT (kg)	% FAT	FFM (kg)	MM (kg)	Soft tissue thickness (cm)	Tissue % FAT	Mean vertebrae area (cm^2^)
Ski Jumping												
r	-0.368	-0.516	-0.322	0.324	-0.001	-0.592	0.762	-0.380	-0.386	-0.529	0.265	-0.512
*p*	*(0*.*070)*	*(0*.*008)*	*(0*.*117)*	*(0*.*114)*	*(0*.*998)*	*(0*.*779)*	*(0*.*717)*	*(0*.*061)*	*(0*,*057)*	*(0*.*007)*	*(0*.*201)*	*(0*.*009)*
Volleyball												
r	-0.036	-0.525	-0.245	0.147	0.069	0.099	0.224	-0.330	-0.329	-0.436	0.180	-0.364
*p*	*(0*.*810)*	*(<0*.*001)*	*(0*.*094)*	*(0*.*317)*	*(0*.*642)*	*(0*.*503)*	*(0*.*125)*	*(0*.*022)*	*(0*.*022)*	*(0*.*002)*	*(0*.*221)*	*(0*.*011)*
Control												
r	0.077	-0.355	0.108	0.356	0.098	0.242	0.277	-0.192	-0.226	-0.149	-0.079	-0.407
*p*	*(0*.*720)*	*(0*.*089)*	*(0*.*617)*	*(0*.*088)*	*(0*.*649)*	*(0*.*255)*	*(0*.*190)*	*(0*.*929)*	*(0*.*916)*	*(0*.*488)*	*(0*.*713)*	*(0*.*049)*
Total												
r	-0.140	-0.559	-0.391	0.068	0.065	-0.072	0.025	-0.468	-0.469	-0463	-0,153	-0.522
*p*	*(0*.*170)*	*(<0*.*001)*	*(<0*.*001)*	*(0*.*507)*	*(0*.*949)*	*(0*.*485)*	*(0*.*810)*	*(<0*.*001)*	*(<0*.*001)*	*(<0*.*001)*	*(0*.*882)*	*(<0*.*001)*

BMI–body mass index; WHtR–waist to height ratio; FAT—Fat mass; %FAT–fat content; FFM–fat free mass; MM–muscle mass

The multiple regression results are presented in Table 4. Four of the body size and composition variables (height, fat mass, muscle mass and soft tissue thickness in L1-L4) explained in total 58.7% of TBS variation. L1-L4 soft tissue thickness made the largest unique contribution to the regression equation (explaining 27% of variation), followed by (in order from largest to smallest) height (14%), fat mass (11%), and muscle mass (6%).

**Table 4:** The results of multiple regression analysis and a unique contribution of predictor variables in TBS variation in all studied subjects.

Variable	Nonstandard coefficients		Standard coefficients			Unique contribution
	Beta	SE	Beta	t	p	U
Constant	2.849	0.159		18.14	<0.001	
Height (m)	-0.563	0.104	-0.86	-5.5	<0.001	0.14
L1-L4 tissue thickness (cm)	-0.042	0.005	-1.08	-7.8	<0.001	0.27
Muscle mass (kg)	0.006	0.001	0.76	3.8	<0.001	0.06
Fat mass (kg)	0.009	0.002	0.55	4.9	<0.001	0.11

F(4,92) = 32.751, p<0.001, R^2^ = 0.587

## Discussion

The key study finding is that soft tissue thickness in the L1-L4 area and stature may affect the calculated indirect texture index of trabecular microarchitecture in the lumbar spine (TBS), at least in male subjects.

According to Silva et al. [18], bone size (which is a derivative of stature) does not affect the TBS, but recent studies suggest otherwise [6, 7]. For instance, a study in women with diverse height indicated that subjects with a lower body stature had higher TBS values than their taller counterparts [6]. Also, in male and female subjects with acromegaly, TBS values were lower compared to controls despite a comparable BMD [7]. Similar relationships were observed in Korean women and men with normal BMD T-score values [19]. In our study, TBS assessment, coming from male subjects diverse in height, also indicated lower than average TBS values in the tallest group (volleyball) compared to shorter athletes (ski jumping) and untrained peers (control). Additionally, values below 1.350, which in adults would be interpreted as degraded or partially degraded microarchitecture [14], were observed in nine volleyball players and six controls, but not in any ski jumpers. Furthermore, a negative relationship of TBS/height was observed both in athletes and the total sample, whereas a negative relationship of TBS/mean vertebra area was visible in all three groups, individually and collectively.

These relationships were verified via regression analyses, together with commonality testing across the total group; all of which identified height as one of the main determinants of TBS. That is, when height increases by 1 cm, the TBS would decrease by 0.006 units on average. To better understand the observed dependence, it is worth considering the technical aspects of DXA image texture processing aimed at extracting microstructural information. The TBS is an index based on quantification of variation in grey-level texture from one pixel to the adjacent pixels, i.e. the rate of grey-level differences between pixels at a specific distance [2, 11, 20]. Thus, a greater height, and hence vertebrae size, increases the number of pixels per vertebra. This means that, in taller subjects, the distance between two pixels represents a smaller relative fraction of the vertebra. Accordingly, and in line with our results, it might be speculated that a lower TBS in very tall individuals reflect erroneous calculation of this texture parameter, but not of worse bone microarchitecture in the lumbar spine. As such, TBS corrections for height seem justified, especially among very tall people, similarly to adjustment of aBMD towards lumbar spine bone mineral apparent density (BMAD), which in this study eliminated the volleyball and ski jumpers group’s difference. This issue requires longitudinal research, because it cannot be ruled out that the disturbed trabecular structure in tall young people could result from rapid bone growth, despite good mineralization. This assumption is supported by results of previous longitudinal studies conducted in young (approx. 13 years old) athletes [21], as well as cross-sectional studies on teenage girls and boys [10], showing that changes in stature are one of the positive predictors of the TBS.

The effect of soft tissue body components on the TBS is poorly understood. Some suggest that the TBS in the L1-L4, as opposed to BMD, is not influenced by the amount of regional soft tissue [22]. However, others reported that the TBS is negatively related to the amount of total fat mass, BMI and other variables measured in the L1-L4, such as trunk fat, visceral fat, trunk lean mass and waist circumference [8, 19, 23–27], as well as the tissue thickness in the measurement area [5, 25]. Our results in lean and very lean male subjects with normal BMI values confirm that TBS is negatively correlated with L1-L4 soft tissue thickness, while it does not depend on BMI, nor does it depend on the amount of fat deposited in the L1-L4 region. Interestingly, in controls, L1-L4 soft tissue thickness was similar to that observed in volleyball players, though it was not associated with TBS. Population differences in tissue composition in the measured region (i.e., a higher percentage of fat and thus a lower lean tissue in controls) offers one explanation. These observations suggest that regional lean tissue, rather than fat content, in the L1-L4 area might affect the TBS. On the other hand, regression analysis identified fat mass as one of four significant predictors of TBS. Previous results [5] suggest that fat and lean tissues might have independent effects on DXA images, due to different absorption characteristics. It is possible that, in the studied athletes, the percentage of fat in the L1-L4 area was so low that the soft tissue thickness in this area became the dominating factor. The influence of individual soft tissues (i.e., fat and lean soft tissue) on the TBS, particularly in the lumbar spine region, not only in male but also female subjects remains to be clarified.

It is also worth emphasizing that, in the literature, only increasing regional soft tissue thickness in L1-L4 area was addressed as a source of potential measurement error (i.e. lower TBS values in obese subjects), while so far, no study has addressed the influence of very low or decreasing soft tissue thickness in this area on the TBS. Meanwhile, among the very slim (lean) ski jumpers, a reduction in L1-L4 soft tissue thickness was linked to higher TBS values, which implies that a minimal amount of tissue in the measurement area can have an opposite effect, leading to overestimation of the TBS. Therefore, it cannot be excluded that the relatively high TBS observed in ski jumping group might reflect a generally thin soft tissue layer.

The present results are in line with suggestions by Kong et al. and Shevroja et al. that using only BMI, as a proxy for the L1-L4 soft tissue thickness, is the weakest component of the current version (3.0.2) of TBS software [1, 25]. This issue is especially pertinent to young subjects, due to the association with changes in body composition, bones, and muscle mass [17]. In the case of physically active populations, consideration of tissue composition in the target area is also justified because, in most of them, BMI does not reflect adiposity due to the larger amount of lean tissue. Therefore, our results suggest the need to modify the current TBS algorithm to account for regional soft tissue thickness [17]. Such a correction appears beneficial due to potentially falsely increased TBS in very lean and slender male subjects. Because the soft tissue thickness in the L1-L4 area is an easily accessible parameter derived from the lumbar spine DXA scan itself, at least in the case of densitometers from two popular manufacturers (i.e. Hologic and GE). Future research concerning tissue analysis in the L1-L4 area in the TBS aspect seems reasonable. Some study limitations should be mentioned; for example, the relatively small sample of solely male subjects limits the generalizability of the reported findings. Moreover, the number of ski jumpers was inadequate to create two separate regression models for each athletic group. Additionally, no direct measure of biological maturity was performed in this study; however, bone age, based on bone alkaline phosphatase activity, provided an index of maturation. Despite the apparent morphological differences, all studied groups presented a similar level of bone development and were of similar chronological age. Furthermore, due to technical limitations of the DXA scanner, we were unable to employ whole body DXA measurement in very tall subjects. To properly analyse the relationships of TBS with body composition (based on the same indicators for all subjects) the BIA method was used to assess body composition. Another limitation is the cross-sectional nature of this work, which made it impossible to account for individual growth rates during puberty, including shifts in body composition, and any impact on the TBS.

In conclusion, among young male individuals with normal BMI, but atypical body build (lean and/or tall), TBS was correlated with body size and composition, mainly with L1-L4 soft tissue thickness, stature and mean vertebra area in lumbar spine, wherein the first two features are the main determinants of TBS variability. Our findings suggest that a very small amount of lean tissue in the L1-L4 area in young male subjects may lead to overestimation of the TBS, while a tall stature may have an opposite effect. Thus, TBS estimates using current software may be partly artefactual and less reflective of the actual status of trabecular bone. It seems that the utility of the TBS could be improved by considering L1-L4 soft tissue thickness as well as stature (or lumbar vertebrae size). There is a need for future research in, morphologically diverse groups, both males and females, to refine the current TBS algorithm and validate it against direct methods (micro-computer tomography or quantitative computer tomography) of trabecular bone structure measurement. Until then, considerable caution is necessary when interpreting TBS, at least in young lean and/or tall subjects.
